# Identification of β4GALNT2 as an anti-hPIV3 factor through genome-wide CRISPR/Cas9 library screening

**DOI:** 10.1080/22221751.2025.2529895

**Published:** 2025-07-16

**Authors:** Xuesheng Wu, Rutger D. Luteijn, Estefanía Lozano-Andrés, Katherine Marougka, Wentao Li, Yoshiki Narimatsu, Frank J. M. van Kuppeveld, Berend Jan Bosch, Robert Jan Lebbink, Erik de Vries, Cornelis A. M. de Haan

**Affiliations:** a Section Virology, Division Infectious Diseases and Immunology, Department Biomolecular Health Sciences, Faculty Veterinary Medicine, Utrecht University, Utrecht, The Netherlands; b Section Immunology, Division of Infection Diseases and Immunology, Department of Biomolecular Health Sciences, Faculty of Veterinary Medicine, Utrecht University, Utrecht, The Netherlands; c Division of Cell Biology, Metabolism and Cancer, Department of Biomolecular Health Sciences, Faculty of Veterinary Medicine, Utrecht University, Utrecht, The Netherlands; d National Key Laboratory of Agricultural Microbiology, Hubei Hongshan Laboratory, College of Veterinary Medicine, Huazhong Agricultural University, Wuhan, People’s Republic of China; e Copenhagen Center for Glycomics, Department of Cellular and Molecular Medicine, Faculty of Health Sciences, University of Copenhagen, Copenhagen, Denmark; f Department of Medical Microbiology, University Medical Center Utrecht, Utrecht, The Netherlands

**Keywords:** Paramyxovirus, CRISPR/Cas9, B4GALNT2, hPIV3, virus entry, sialic acid

## Abstract

Human respirovirus 3 (also known as human parainfluenza virus 3; hPIV3) is a major cause of severe acute respiratory infections in vulnerable populations. Here we conducted a genome-wide CRISPR/Cas9 library screen to identify key host factors for hPIV3 infection. In addition to identifying several host proteins involved in glycosylation as proviral factors, we identified β-1,4-N-Acetyl-Galactosaminyltransferase 2 (β4GALNT2) as a potent restriction factor. Further investigation demonstrated that the addition of a GalNAc residue to α2-3-sialylated glycans by β4GALNT2, resulting in the Sd^a^ glycotope, disrupted the interaction between the viral hemagglutinin-neuraminidase (HN) attachment protein and sialoglycan receptors. Specifically, the additional GalNAc residue interfered with the interaction of residue W371 in HN with sub-terminal glycan moieties. β4GALNT2-mediated Sd^a^ epitope expression also negatively affected infection by other respiroviruses, with the strongest effect being observed for hPIV3.

## Introduction

Paramyxoviruses (PMVs) with an haemagglutinin-neuraminidase (HN) glycoprotein initiate infection by binding to α2-3-linked sialic acid (2-3Sia) receptors on the plasma membrane [1]. In addition to Sia-binding, HN also functions to cleave Sia receptors, allowing virion mobility [2,3] and progeny virion release [4]. An optimal balance between Sia-binding and – cleavage functions of HN is essential for efficient viral replication [2,5–7].

Although it is well established that 2-3Sia act as entry receptors for PMV [1,8–10], details of receptor fine specificity, including modifications on Sia itself or subterminal glycans remains unclear. Glycan array analysis showed that the Siaα2-3Galβ1-4GlcNAc glycotope is the primary glycan structure bound by human respirovirus 1 and 3 (also known as human parainfluenza virus 1 and 3, hPIV1 and hPIV3) [8,9], but whether these also function as entry receptors has not been established. In addition, it is not known whether specific proteins play a role in PMV entry, similarly as has been reported for some sialoglycan-binding influenza A viruses (reviewed in [11]).

CRISPR/Cas9 screens have been applied to various viruses, such as influenza virus (reviewed in [12]) and coronaviruses [13], to identify host factors relevant to virus infection. A CRISPR/Cas9 screen identified several innate immunity-related restriction factors for hPIV3 [14], in agreement with the known antiviral role of such factors in PMV replication (reviewed in [15]). CRISPR/Cas9 screens also identified several proviral genes involved in glycosylation and Sia metabolism for hPIV3 [14] and SeV [16], deletion of which inhibited infection. However, specific host factors critical for PMV entry beyond Sia have not been reported. A notable challenge in using CRISPR/Cas9 to identify host factors is that PMVs generally do not induce apoptosis in certain cells [17–20], complicating the enrichment of infected versus uninfected cells.

In this study, we used a hPIV3 strain expressing green fluorescent protein (GFP), combined with fluorescence-activated cell sorting (FACS) to enrich specific cell populations, leading to the identification of several genes involved in the generation of 2-3Sia-containing glycans act as proviral factors. In addition, we elucidated, β4GALNT2 (β-1,4-N-Acetyl-Galactosaminyltransferase 2) to be an antiviral factor. β4GALNT2 modifies 2-3Sia containing glycans therefore preventing infection by several PMVs, which was most pronounced for hPIV3.

## Results

### Genome-wide crispr/cas9 screen reveals host factors regulating hPIV3 infection

**Figure 1.**
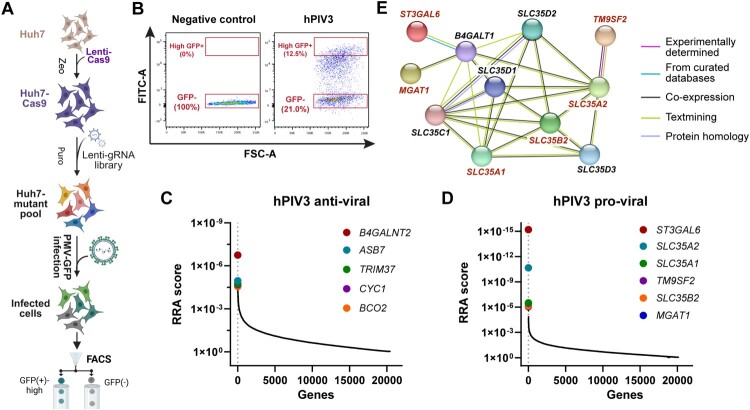
Genome-wide CRISPR/Cas9-based genetic screens in Huh7 cells identifying host factors for hPIV3 infection. (A) Schematic of the CRISPR/Cas9 screen workflow in Huh7 cells. Cas9-expressing Huh7 cells were transduced with lentiviral human sgRNA libraries, then infected with GFP-tagged hPIV3 at multiplicity of infection (MOI) 10 for 20 h. Infected cells were sorted by fluorescence-activated cell sorting (FACS) based on GFP expression to isolate high-GFP or GFP-negative populations. Genomic DNA (gDNA) was extracted and sgRNA sequences were amplified, followed by PCR and sequencing. (B) Gating strategy of hPIV3-infected Huh7-mutant pool, the high-GFP expressing or low-GFP expressing cells were enriched accordingly. Negative control are non-infected cells. (C and D) CRISPR screen results for anti- (C) and pro-(D)-viral genes in Huh7 cells. Genes were ranked by robust rank aggregation (RRA) scores, calculated using MAGeCK [25]. Lower RRA scores in panels C and D indicate antiviral and proviral genes, respectively. (E) STRING network [26] analysis of the top-6 proviral genes (highlighted in red). Genes shown in black were automatically added by selecting the “add more nodes” option to connect the input genes. Colours represent the types of interactions according to the legend. The functions of genes listed in C and D are listed in Table S2.

To identify host factors influencing hPIV3 infection, we performed a genome-wide CRISPR/Cas9 knock out screen using mutagenized Huh7 cells (Huh7-mutant pool) (Figure 1A) that had been generated and successfully used previously [21]. Prior to infection, flow cytometry analysis confirmed high levels of mCherry expression following puromycin selection, indicating efficient incorporation of sgRNA constructs (Fig. S1). These cells, transduced with a comprehensive CRISPR/Cas9 library, were challenged in a single round infection assay with GFP-tagged hPIV3 at a high multiplicity of infection (MOI = 10) for 20 hours (Figure 1A). Post-infection, fluorescence-activated cell sorting (FACS) was used to isolate GFP-high (infected) and GFP-negative (virus-resistant) cell populations (Figure 1B and Fig. S2). Virus-sensitive or -resistant cells were collected and abundance of single-guide RNA (sgRNAs) in each population was determined by Illumina sequencing. Comparing the enriched sgRNAs between virus-sensitive and virus-resistant cells enabled the identification of genes with either antiviral (*B4GALNT2, ASB7, TRIM37, CYC1, BCO2*; sgRNAs enriched in GFP-high cells) (Figure 1C) or proviral (*ST3GAL6, SLC35A2, SLC35A1, TM9SF2, SLC35B2, MGAT1*; sgRNAs depleted in GFP-high cells) (Figure 1D) functions (Table S1). The proviral genes are all involved in glycosylation (Table S2), in agreement with the known role of sialoglycans in hPIV3 entry [2,7,22,23], and functionally associated with each other as demonstrated by String network analysis (Figure 1E), thereby validating the screen. Among the antiviral factors, the strongest hit was the glycosyltransferase gene *B4GALNT2* encoding the Sd^a^ synthase [24].

## *B4GALNT2* overexpression inhibits hPIV3 infection

**Figure 2.**
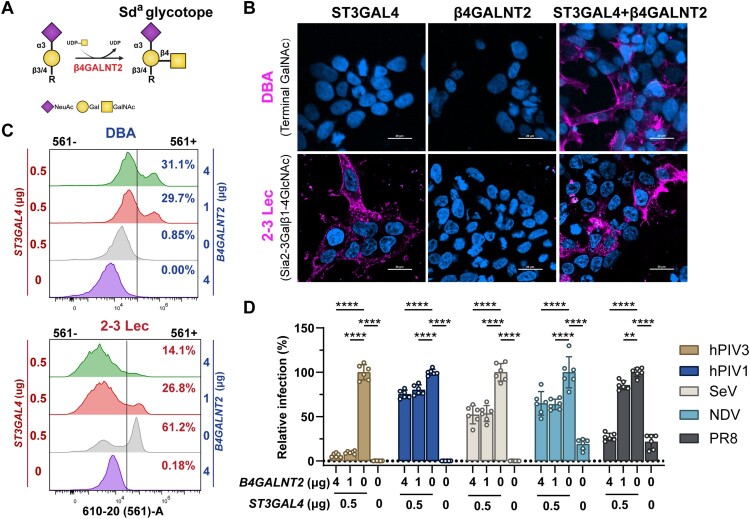
Expression of Sd^a^ antigen by *B4GALNT2* inhibits hPIV3 infection. (A) Schematic representation of the glycan modification catalyzed by β4GALNT2. Glycan symbols are drawn according to the SNFG format [34]. (B) Fluorescently labelled DBA or 2–3 Lec were applied to HEK^ΔSia^ cells co-transfected with ST3GAL4 (0.5 μg) or β4GALNT2 (4 μg)-encoding plasmids, either independently or in combination. Scale bar = 20 μm. (C) Flow cytometry histogram overlays showing the binding of the specific lectins to HEK^ΔSia^ cells co-transfected with varying amount of ST3GAL4 and β4GALNT2-encoding plasmids. Dolichos biflorus agglutinin (DBA), recognize terminal GalNAc; 2–3 Lectenz (2-3 Lec) recognizes 2–3 sialylated N-glycans. (D) Flow cytometry analysis of different virus infection efficiency (MOI = 2) in HEK^ΔSia^ cells co-transfected with varying amount of ST3GAL4 and β4GALlNT2-encoding plasmids. Data are normalized to the infection level of HEK^ΔSia^ cells transfected with only ST3Gal4-encoding (0.5 μg) plasmid. The bars represent the mean of two independent experiments, each performed in triplicate. The flow cytometry analysis gating strategy is presented in Fig. S3. Data are presented as mean ± SD. * *P*≤0.05, ** *P*≤0.01, *** *P*≤0.001 and **** *P* < 0.0001.

The β4GALNT2 enzyme catalyzes the transfer of a N-acetyl-galactosamine (GalNAc) residue to α2-3-sialylated disaccharides (Neu5Acα2-3Galβ1-3/4-R) found either on O-glycans, glycolipids, or N-glycans, forming the Sd^a^ histoblood group antigen (GalNAcβ1-4[NeuAcα2-3]Galβ1-R) (Figure 2A) [27,28]. To investigate the role of β4GALNT2 in hPIV3 infection in more detail, we made use of HEK293 cells, which naturally lack the Sd^a^ epitope (Figure 2B and 2C). [29,30]. Sia-deficient HEK293 cells (HEK^ΔSia^), which lack all β-galactoside sialyltransferase [30], were transfected with varying concentrations of β4GALNT2-encoding plasmids in the absence or presence of plasmid encoding ST3 β-galactoside α2-3 sialyltransferase 4 (ST3GAL4), which catalyzes the transfer of Sia to galactose-containing glycans in an α2-3 linkage to Galβ1-4GlcNAc (LacNAc) terminal disaccharides [31]. Sd^a^ epitope expression and α2,3-linked sialic acid (2-3Sia) levels were assessed using Dolichos biflorus agglutinin (DBA) lectin and 2–3 Lectenz (2-3 Lec) staining, respectively (Figure 2B-C). DBA primarily recognizes the α-linked N-acetyl galactosamine (GalNAc), which in HEK293 cells is only produced when β4GALNT2 in introduced to a 2-3Sia glycan and therefore only observed upon co-expression of *ST3GAL4*, while 2–3 Lec specifically recognizes the Siaα2-3Galβ1-4GlcNAc glycotope. Increasing *B4GALNT2* concentrations reduced 2–3 Lec staining, indicating that the Sd^a^ epitope interferes with 2–3 Lec recognition (Figure 2C), consistent with prior findings [32,33]. Notably, Sd^a^ epitope expression severely inhibited hPIV3 infection (Figure 2D), and showed a milder, yet significant, inhibitory effect on hPIV1, Sendai virus (SeV) and Newcastle disease virus (NDV) infections. Influenza A virus H1N1 Puerto Rico/8 (PR8), previously reported [32,33] to be inhibited by *B4GALNT2* expression, was used as a positive control. Overexpression of *B4GALNT2* alone did not affect infection for the four tested viruses in HEK^ΔSia^ cells (Fig. S4). These findings demonstrate that β4GALNT2-mediated Sd^a^ epitope expression variably restricts PMV infection, with the strongest effect observed for hPIV3.

## Sd^a^ antigen reduces paramyxovirus-sialoglycoprotein binding

**Figure 3.**
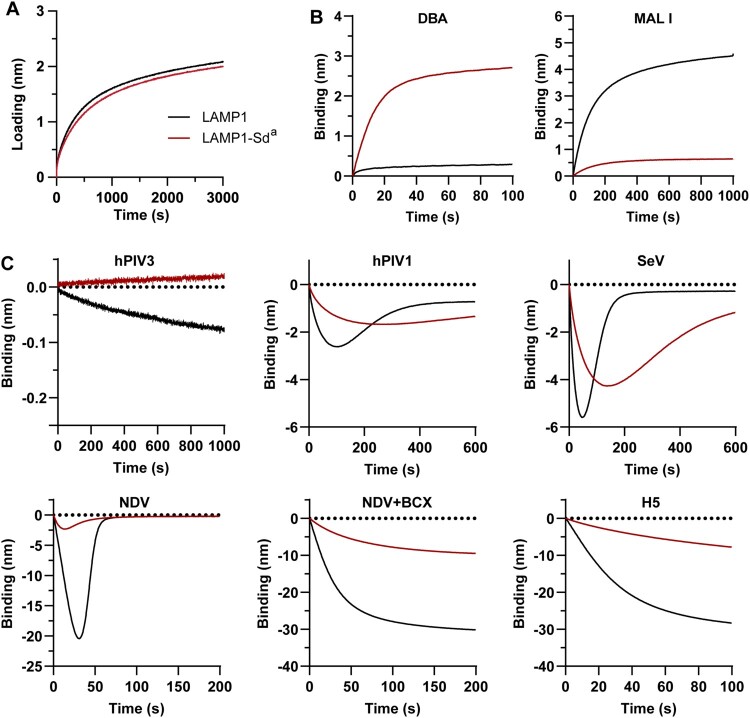
Sd^a^ antigen expression reduces paramyxovirus HN and influenza HA binding to glycoprotein receptors. (A) LAMP1 and LAMP1-Sd^a^ glycoproteins were loaded onto the BLI sensor at comparable levels. (B) Lectin characterization of LAMP1 and LAMP1-Sd^a^ using DBA and MAL I. MAL I recognizes Neu5Acα2-3Galβ1-4GlcNAc (a trisaccharide common in N-glycans). (C) BLI analysis to determine the binding kinetics of nanoparticles displaying paramyxovirus HN or influenza HA glycoproteins to LAMP1 and LAMP1-Sd^a^. Binding was assessed using an established HN-Ni NTA nanoparticle (HN-NPs) system using 3.5×10^10^ HN-NPs or HA-NPs per well. Binding of NDV HN-NPs was additionally tested in the presence of site-I specific inhibitor BCX2798 (BCX). Each experiment was conducted at least twice, with representative results displayed here.

To validate the antiviral effect of the Sd^a^ antigen, we performed virus-glycoprotein binding assays using glycoproteins with or without Sd^a^ epitope decoration. To this end, the luminal domain of lysosomal-associated membrane glycoprotein I (LAMP1) was expressed and purified from HEK^ΔSia^ cells expressing *ST3GAL4*, with or without additional co-expression of *B4GALNT2*, to introduce Sd^a^ epitopes (LAMP1-Sd^a^). The purified glycoproteins were subsequently loaded onto the BLI streptavidin sensor at comparable levels (Figure 3A). The presence of the Sd^a^ antigen on LAMP1 was confirmed by DBA lectin binding, which also showed a corresponding reduction in Maackia amurensis lectin I (MAL I) binding, specific for Neu5Acα2-3Galβ1-4GlcNAc epitopes (Figure 3B). Since the GalNAc is only incorporated into sialylated structures, the overall sialylation level of LAMP1-Sd^a^ is expected to match that of LAMP1. Next, we assessed virus-glycoprotein interaction using biolayer interferometry (BLI) and a nanoparticle (NP) system displaying viral PMV HN or IAV HA glycoproteins (HN/HA-NPs) [7]. Influenza H5 infection, previously shown to be inhibited by Sd^a^ antigen [33], was used as a positive control. Notably, in BLI analyses of particle-receptor interactions, a negative binding curve is observed, with stronger binding being reflected in larger negative values [2,7,35,36]. NPs decorated with the viral glycoproteins displayed differential binding levels in agreement with previous results [7]. BLI analysis demonstrated that Sd^a^ glycotope expression eliminated hPIV3 HN-NP binding to LAMP1 (Figure 3C). For other respiroviruses (hPIV1 and SeV) and NDV, the Sd^a^ glycotope also reduced HN-NP binding to LAMP1 (Figure 3C), but did not fully abolish it, similarly to influenza H5. Further bending of the curves after reaching peak binding, observed for HN-NPs but not HA-NPs, corresponds to the release of HN-NPs from the sensor surface, suggesting that Sia of the Sd^a^ glycotope can be cleaved by HN. To further dissect the interaction between NDV HN’s secondary Sia binding site (Site II, not functionally present in respirovirus HN) and the Sd^a^ glycotope, we included the primary binding site (Site I)-specific inhibitor BCX2798 (4-azido-5-isobutyrylamino-2,3-didehydro-2,3,4,5-tetradeoxy-d-glycero-d-galacto-2-nonulopy-ranosic acid, BCX) [2,7] to selectively block Site I. Binding mediated by Site II was also inhibited, albeit not completely, by the presence of the Sd^a^ glycotope. In conclusion, β4GALNT2 acts as a restriction factor for PMVs, particularly hPIV3. The Sd^a^ glycotope significantly interferes with PMV-sialoglycan binding (Figure 3), thereby reducing viral infection (Figure 2D).

## Sd^a^ antigen disrupts hPIV3 HN interaction with penultimate and antepenultimate glycan residues

**Figure 4.**
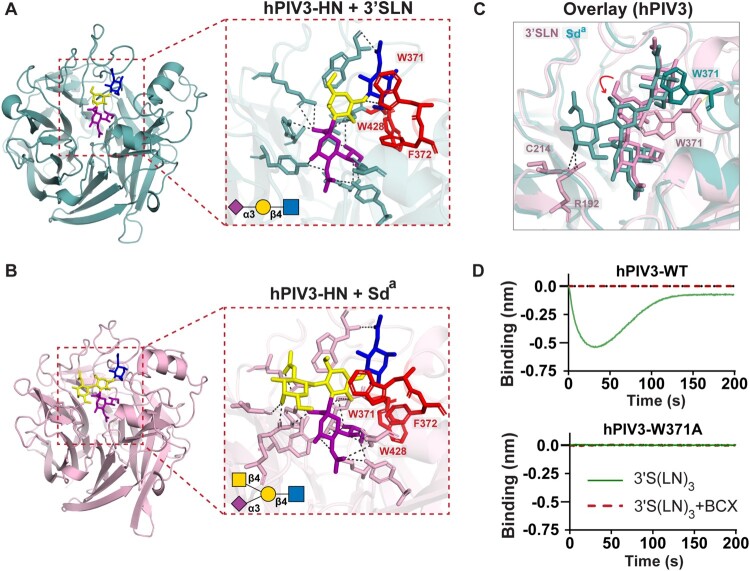
Structural basis for Sd^a^-mediated disruption of hPIV3 HN-glycan interactions. (A, B) Predicted interactions between hPIV3 HN monomer and ligands using Chai Discovery (https://lab.chaidiscovery.com) [22]. Panel (A) illustrates the binding of HN to 3′-sialyl-N-acetyllactosamine (3'SLN), while panel (B) depicts the interaction with the Sd^a^ glycotope. Aromatic residues W371, F372 and W428 are shown in red. (C) Overlay of the predicted interactions, highlighting the differences between the two ligands. The interaction between GalNAc and residues C214 and R129 induces a shift in the entire glycan chain, as indicated by the red arrow. (D) Binding analysis of hPIV3 HN wild-type (WT) and W371A mutant HN-NPs to 3'S(LN)_3_ was conducted using BLI, in the presence or absence of the BCX2798 inhibitor, using 3.5×10^10^ HN-NPs per well. The difference in the binding curve for hPIV3 HN-NPs compared to Figure 3C results from the different receptors (LAMP1 vs 3’S[LN]_3_) being used. All experiments were performed independently in triplicate, with representative data presented.

To gain structural insight into the antiviral mechanism of the Sd^a^ glycotope, we employed Chai Discovery [37], an artificial intelligence platform designed to predict interactions between biochemical molecules, to analyze the binding between the hPIV3 HN protein and sialoglycans, specifically 3′-sialyl-N-acetyllactosamine (3′SLN) and the Sd^a^ glycotope (Figure 4A to C). Analysis of the HN-3′SLN interaction revealed that the nitrogen atom of W371 forms a hydrogen bond with the oxygen atom of the glycosidic bond between Gal-2 and GlcNAc-3. Aromatic residues F372 and W428 further stabilize the glycan chain by stacking with W371, anchoring the 3′SLN trisaccharide within the HN binding pocket (Figure 4A). In contrast, the presence of the Sd^a^ glycotope disrupts this interaction. The additional GalNAc residue establishes new contacts with residue C214, inducing a shift in the glycan chain (Figure 4B). This shift disrupts the hydrogen bonds between W371 and the oxygen atom of the glycosidic bond between Gal-2 and GlcNAc-3, destabilizing the interaction (Figure 4C). These findings align with an earlier glycan array study, which demonstrated binding of hPIV3 to Siaα2-3Galβ1-4GlcNAc-R but not to variants containing the Sd^a^ glycotope (Fig. S5) [9]. To confirm the important role of W371 in receptor binding, we generated an HN mutant (W371A) and assessed its binding to the 3′S(LN)_3_ receptor using HN-NPs and BLI. Mutation of W371 to alanine abolished HN-NP binding to the receptor (Figure 4D). These findings show that the stabilization of HN-sialoglycan interactions relies on residues beyond those directly interacting with Sia-1. The Sd^a^ antigen disrupts these interactions, particularly those involving W371 with the oxygen atom of the glycosidic bond between Gal-2 and GlcNAc-3, thereby inhibiting hPIV3 receptor binding and infection.

## Discussion

Using CRISPR/Cas9 knockout technology combined with FACS-based enrichment, we identified host genes that either promote or restrict hPIV3 infection. The setup of our screen (single-round infection using a GFP-reporter virus) particularly resulted in hits involved in early-stages of infection and highlight the crucial role of glycosylation in hPIV3 receptor engagement and entry. In particular, the proviral role of several solute carrier family genes (SLC35), *MGAT1* and *ST3GAL6* (Table S2) [16,27,28,30,38–49] points towards an important role of sialylated N-glycans, in agreement with the known importance of Sia for hPIV3 attachment and entry [2,7,16,22,23]. Notably, *SLC35A1* and *SLC35A2* emerged as top proviral genes, consistent with recent CRISPR knockout screen on SeV [16], where glycosylation-related genes were also prominently enriched. In contrast, β4GALNT2, which is responsible for the generation of the Sd^a^ glycotope, emerged as the strongest inhibitory factor against hPIV3 infection, with the Sd^a^ glycotope interfering with receptor binding and subsequent infection. The antiviral roles of other hits we identified (Table S2) remain to be confirmed. The absence of a prominent role for genes from the interferon signalling pathway in our screen is likely due to the early time point analyzed. Other limitations of the screen are the use of Huh7 cells rather than differentiated epithelial cells and of a lab-adapted hPIV3 virus. In future studies it will therefore be important to validate the role of identified hits in more physiologically models, such as primary human air-to-liquid interphase models in combination with clinical strains.

B4GALNT2, not only suppressed infection by hPIV3, but also that of multiple other PMVs, including hPIV1, SeV and NDV. B4GALNT2 was previously also identified as an inhibitor of influenza virus infection [32,33]. The inhibitory effect of the Sd^a^ glycotope on hPIV3 infection aligns with a prior glycan array study (Fig. S5) [9], in which no binding was observed for hPIV3 to glycans carrying this glycotope. In contrast, hPIV1 was shown to bind, in agreement with a smaller negative effect of the Sd^a^ glycotope on infection (Figure 2D) and binding (Figure 3C) with this virus. For NDV, β4GALNT2 was recently also identified as an antiviral factor, although its effect was more limited as compared to avian IAV infection [50], in agreement with our results. The larger negative effect of the Sd^a^ glycotope on hPIV3 infection compared to other PMVs may be linked to its low binding (Kd ∼4 mM) enzyme affinity (K_m_ = ∼1 mM) [7,39] and reduced overall binding as assessed by BLI ([2,7] and this paper). Consequently, hPIV3 not only binds to lower levels, but also requires a longer time to for release resulting from receptor destruction. Reduced receptor binding may subsequently also negatively affect fusion protein activation and membrane fusion [1,40–42].

Structural prediction of hPIV3 HN-sialoglycan interaction revealed that adding GalNAc to the penultimate Gal-2 disrupts the interactions of residue W371 with sub-terminal glycan moieties Gal-2 and GlcNAc-3, probably reducing the affinity below a threshold level needed for efficient cell binding and fusion. The importance of W371 in receptor binding was confirmed by analyzing a W371A mutant HN protein. In agreement herewith, crystal structures of mumps virus (MuV) HN reveal that residue Y369 (analogous to W371 in hPIV3) stabilizes interactions of HN with sub-terminal glycan residues [10].

The Sd^a^ histoblood group epitope is present on erythrocytes, certain organs and body secretions, and has been implicated in various biological processes, including hemostasis, muscular dystrophy, reproduction, and intestinal microbiota regulation (reviewed by [24]). The expression level of *B4GALNT2* has been proposed as a prognostic marker for colon and breast cancer and its manipulation might represent a therapeutic perspective for colon cancer and muscular dystrophy. A small subset of the human population lacks the Sd^a^ antigen without apparent pathological consequences. Here we identified β4GALNT2 as a restriction factor for hPIV3 and other PMVs. The Sd^a^ antigen, installed by β4GALNT2, inhibits PMV HN binding to sialoglycoprotein by disrupting interactions with subterminal glycan residues. Possibly, differences in Sd^a^ expression may also affect PMV infection dynamics and disease severity *in vivo*. Additionally, as mentioned earlier [33], B4GALNT2 may be an attractive host factor to target therapeutically.

## Material and methods

### Cells and reagents

Human hepatoma (Huh7) cells, Sia-deficient human embryonic kidney 293 (HEK^ΔSia^, by knockout of *ST3GAL1-6* and *ST6GAL1/2* genes) cells [30] were cultured in Dulbecco’s modified Eagle’s medium (DMEM) (Thermo Fisher Scientific), supplemented with 10% fetal bovine serum (Biowest), 1 mM sodium pyruvate (Gibco), 100 IU/ml penicillin, and 100 IU/ml streptomycin (Lonza), at 37°C in a humidified CO_2_ incubator. All cell lines were regularly screened and confirmed negative for mycoplasma contamination. BCX2798 (4-azido-5-isobutyrylamino-2,3-didehydro-2,3,4,5-tetradeoxy-D-glycero-D-galacto-2-nonulopy-ranosic acid) were synthesized in house as described previously [2].

NDV (Nobilis ND Clone-30) was purchased from MSD Animal Health and used directly in experiments. The GFP-expressing strains of hPIV1 (P121), hPIV3 (P323) and SeV (S124) were purchased from ViraTree. These viruses were created based on the following strains: hPIV1 (strain Washington/20993/1964, GenBank accession no. AF016280), hPIV3 (strain JS, GenBank accession no. KY295925) and SeV (strain Z, GenBank accession no. M30202). hPIV1, hPIV3 and SeV were propagated in MDCK-II cells using Opti-MEM (Thermo Fisher Scientific). 1 μg/ml TPCK trypsin (Sigma-Aldrich) was additionally added for hPIV1 and SeV. The viruses were aliquoted and stored at −80 °C until use. The virus titres of the different virus preparations were determined by 50% tissue culture infectious dose (TCID_50_) assay on MDCK-II cells based on GFP expression for hPIV1, hPIV3 and SeV, and by fluorescence staining of NDV-infected cells (Abcam, ab34402) as readout.

## Genome-wide crispr/cas9 library screen

A Huh7-mutant pool incorporating a genome-wide CRISPR/Cas9 library, specifically designed to target all protein-encoding genes in the human genome, was previously generated [21]. This library contains ±260,000 unique sgRNAs, with each gene being targeted by ±10-12 sgRNAs [21]. Prior to viral infection, over 95% of the cells were confirmed to express the mCherry marker encoded by the lentiviral sgRNA library vector. To determine the optimal multiplicity of infection (MOI) for screening, GFP expression was assessed at 20 hours post-infection (h.p.i.) and an MOI of 10 was selected. Approximately 150 million sgRNA-positive cells were infected with hPIV3-GFP at MOI 10. At 20-h.p.i., the cells were washed with Opti-MEM (Thermo Fisher Scientific) and detached using trypsin-EDTA (Gibco, USA). The cells were then collected by centrifugation at 1500 g for 3 minutes, washed twice with Opti-MEM, and diluted to a concentration of 10 million cells/ml on ice. Fluorescence-activated cell sorting (FACS) was performed using a FACS Aria Fusion (Becton Dickinson) equipped with an 85 nozzle and installed within a flow hood. mCherry signal was measured from the 561 nm laser using a 582/15 bandpass filter, while GFP signal was measured from the 488 nm laser using a 530/30 bandpass filter and cell populations were separated as GFP-high (infected) and GFP-negative (virus-resistant) using a 2-way purity mode and proceeded at speeds up to 6000 events per second. The sorted cell populations were subjected to deep sequencing analysis as described previously, using the Model-based Analysis of Genome-wide CRISPR/Cas9 Knockout (MaGeck) pipeline [43]. The average distribution of the gRNAs in the preselected cells had a Gini-coefficient 0.28, which is slightly higher than the recommended Gini-coefficient of <0.2. 10% of the gRNAs were missing in the preselected population. The quality of the data was sufficient for the identification of high-confidence hits. However, the identification of other hits, including potential (redundant) protein receptors may have required a dataset with a gRNA higher coverage and better gRNA distribution. Flow cytometry data files were exported from BD FACSDiva and analyzed using FlowJo V10 (FlowJo, Ashland, USA).

## Lectin staining to quantify the glycosylation profile

HEK^ΔSia^ cells were seeded onto 12 mm glass coverslips in 24-well plates (5 × 10^4^ cells/well, Corning Costar). The following day, cells were transfected with the indicated plasmids using FuGene 6 (Promega, E2691). To ensure consistent total DNA amounts during transfection, empty pcDNA vector was added to balance plasmid quantities as needed. At 48 hours post-transfection, cells were fixed with 3.7% paraformaldehyde (PFA) in PBS^+/+^ (Pate-buffered saline with Ca^2+^ and Mg^2+^, Lonza), then blocked for 1 hour with high-purity BSA (Sigma, A7638). Cells were incubated on ice for 1 hour with biotinylated lectins (SiaFind Alpha 2,3-Specific Lectenz [2-3 Lectenz; 2–3 Lec] from Lectenz Bio, or DBA from Vector Labs) in PBS^+/+^. Afterward, they were stained for 1 hour with Alexa Fluor 568-conjugated streptavidin (Thermo Fisher) in PBS^+/+^. Confocal immunofluorescence microscopy (Nikon A1R) was used to examine the cells. For flow cytometry measurements, cells were detached 48 hours post-transfection using Cell Dissociation Buffer (Gibco) and subjected to the same lectin staining protocol. Data acquisition was performed on a CytoFLEX LX flow cytometer (Beckman Coulter), and results were analyzed using CytExpert software.

## Quantification of virus infection by flow cytometry

HEK^ΔSia^ cells were seeded in 24-well plates (1.0 × 10^5^ cells/well) and the next day transfected using FuGene 6 with specific plasmids as indicated in the text. At 48- h.p.t, cells were infected with virus at MOI at 2. Cells were collected at 24-h.p.i and fixed with 3.7% PFA. For the GFP-expressing viruses (hPIV1, hPIV3 and SeV), cells were directly subjected to flow cytometry analysis after fixation. For NDV, cells were stained by incubation with anti-NDV antibodies (Abcam, ab34402) followed by goat anti-chicken antibodies (Alexa FluorTM 488, Thermo, A11039). Subsequently, cells were subjected for flow cytometry analysis using a CytoFLEX LX (Beckman Coulter). The gating methods are based on standard protocols [44] and a representative flow cytometry gating strategy for virus-infected cells is shown in Fig. S3.

## Recombinant glycoprotein expression and purification

Recombinant viral proteins were produced in Freestyle 293 cells (293F, Gibco) cultured in Freestyle 293 expression medium at 37°C. Human codon-optimized cDNAs encoding the ectodomains of HN proteins from hPIV1 (GenBank: AAC23946.1), hPIV3 (GenBank: AAB48689.1), SeV (GenBank: P0845.3), and NDV (GenBank: CAB51326.1) were synthesized by GenScript. These cDNAs were inserted into the pFRT expression plasmid (Thermo Fisher Scientific) containing an exogenous CD5 signal peptide, a 6×Histidine tag, a Twin-Strep tag, and a tetrabrachion tetramerization domain. The HN proteins were transiently expressed in 293F cells, and the secreted proteins were purified from the culture supernatants using Strep-Tactin beads (IBA) according to the manufacturer’s instructions.

Human codon-optimized cDNAs encoding the ectodomains of LAMP1 (GenScript) were cloned into the pCAGGS vector (*pCAGGS-LAMP1*), incorporating a C-terminal Twin-Strep tag, a biotin-acceptor peptide (BAP; GGLNDIFEAQKIEWH) for site-specific biotinylation by BirA, and a 6xHis tag for purification using Ni-NTA beads [45]. Biotinylated LAMP1 was expressed in HEK^ΔSia^ cells co-transfected with an expression vector encoding the BirA biotin ligase with a CD5 signal sequence (*pCD5-BirA*) [45]. To modify the sialylation patterns of secreted recombinant glycoproteins, expression vectors encoding ST3 β-galactoside α-2,3-sialyltransferase 4 (*pcDNA-ST3GAL4*; GenScript) and *pcDNA-B4GALNT2* (GenScript) were co-transfected with *pCAGGS-LAMP1* and *pcD5-BirA* into HEK^ΔSia^ cells using polyethyleneimine (PEI, Polysciences) as previously described [45]. Secreted proteins were collected from the culture supernatants at 84 hours post-transfection and purified using Ni-NTA beads according to the manufacturer’s protocol (Thermo Fisher Scientific, K95001).

## Preparation of HN-Ni NTA nanoparticles (HN-NPs)

The preparation of HN-Ni NTA nanoparticles (HN-NPs) follows the protocol established in our previous study [7]. Dextran iron oxide composite Ni-NTA nanomag-D nanoparticles (130 nm) were obtained from Micromod. The standard coupling ratio used was 0.45 μg of HN protein per 7.43 × 10^8^ nanoparticles, with particle counts determined via nanoparticle tracking analysis, in a total volume of 100 µl PBS^+/+^. Coupling was performed overnight at 4°C with constant shaking. Following incubation, the HN-NPs were centrifuged at 2,000 rpm for 10 minutes, and the supernatant was carefully removed to eliminate unbound proteins. The pellet was resuspended in an equal volume of PBS^+/+^. Details regarding the number of HN-NPs used for BLI analysis are provided in the legends.

## Biolayer interferometry analysis (BLI)

PBS^+/+^ was used as the standard buffer for all BLI assays. Standard streptavidin (SA, Pall-ForteBio) biosensors were employed for BLI “dip and read” analyses, following a similar procedure as described previously [35]. Briefly, the SA sensors were loaded with specific biotinylated LAMP1 glycoproteins and incubated in PBS^+/+^ until a stable baseline was achieved. The receptor-loaded sensors were then exposed to PBS^+/+^ containing HN-NPs or lectins for a specified period to generate binding curves, with 0.5 mM BCX2798 [2] included where indicated. Sensor regeneration was achieved through three 5-second washes in 10 mM Tris/Glycine buffer (pH 2.0), which removed bound HN-NPs or lectins while preserving the biotinylated receptor [2]. All experiments were conducted at 30°C. The observed negative binding curves are attributed to the large size of the nanoparticles relative to soluble proteins, a phenomenon consistent with findings involving enveloped virions and vesicle-like structures [2,7,36,46,59].

## Supplementary Material

Table S2.docx

Fig S2.tif

Fig S3.tif

Figure S5 reprint permit.pdf

Fig S1.tif

Fig S4.tif

Table S1.xlsx

Biorender Publication License.pdf

Fig S5.tif
