# Supplementary material for: Identification of β4GALNT2 as an anti-hPIV3 factor through genome-wide CRISPR/Cas9 library screening
Source: Emerg Microbes Infect. 2025 Jul 16;14(1):2529895. doi: 10.1080/22221751.2025.2529895 (PMC12269090; doi:10.1080/22221751.2025.2529895)
Supplement: Table S2.docx [file TEMI_A_2529895_SM5467.docx]

## Table S2. Functions of antiviral and proviral genes shown in Fig. 1C and D.

| **Genes** | **Anti- or Pro-viral** | **Function** | **Ref.** |
| --- | --- | --- | --- |
| *B4GALNT2* | Antiviral | Catalyzes the final step in the biosynthesis of the human Sd^a^ glycotope by adding a GalNAc residue through a β-1,4 linkage to a subterminal galactose residue that is substituted with an ⍺-2,3-linked sialic acid. | [27,28] |
| *ASB7* | Antiviral | A likely substrate-recognition component of an SCF-like ECS (Elongin-Cullin-SOCS-box protein) E3 ubiquitin-protein ligase complex, facilitating the ubiquitination and subsequent proteasomal degradation of target proteins. | [48] |
| *TRIM37* | Antiviral | - E3 ubiquitin-protein ligase required to prevent centriole reduplication - Probably acts by ubiquitinating positive regulators of centriole reduplication - Mediates monoubiquitination of 'Lys-119' of histone H2A (H2AK119Ub), a specific tag for epigenetic transcriptional repression - associates with some Polycomb group (PcG) multiprotein PRC2-like complex and mediates repression of target genes | [49,50] |
| *CYC1* | Antiviral | - Functions as a component of ubiquinol-cytochrome c oxidoreductase, facilitating the transfer of electrons from the Rieske Fe-S protein to cytochrome c. - Serves as a subunit of mitochondrial complex III, a key component of the mitochondrial respiratory chain and a significant source of reactive oxygen species (ROS) generation under both physiological and pathological conditions. | [51] |
| *BCO2* | Antiviral | - A broad-specificity mitochondrial dioxygenase that catalyzes the asymmetric oxidative cleavage of carotenoids. - Cleaves carotenes (hydrocarbon carotenoids), including all-trans-beta-carotene and lycopene, as well as xanthophylls (oxygenated carotenoids), such as zeaxanthin, lutein, and beta-cryptoxanthin, at both the 9,10 and 9',10' carbon-carbon double bonds. - Plays a critical role in carotenoid metabolism, contributing to the regulation of oxidative stress and the production of key signaling molecules. | [52] |
| *ST3GAL6* | Proviral | Transfers sialic acid residues in an ⍺-2,3 linkage to terminal galactose on glycoproteins and glycolipids. | [53,54] |
| *SLC35A2* | Proviral | - Transports uridine diphosphate galactose (UDP-galactose) from the cytosol into the Golgi apparatus. - Functions as an antiporter, exchanging UDP-galactose for UMP. - Exchanges UDP-galactose for AMP and CMP. - Transports UDP-N-acetyl galactosamine (UDP-GalNAc) and other nucleotide sugars. - Supplies UDP-galactose to galactosyltransferases in the Golgi apparatus. - Essential for the synthesis of globotriaosylceramide/ globoside (Gb3Cer) from lactosylceramide. | [16,55] |
| *SLC35A1* | Proviral | - Transports CMP-sialic acid from the cytosol into the Golgi apparatus. - Functions as an antiporter, exchanging CMP-sialic acid for CMP. | [55,56] |
| *TM9SF2* | Proviral | - May function as a small molecule transporter or as an ion channel. - TM9SF2 deficiency causes disruption in glycosphingolipid biosynthesis - TM9SF2 deficiency reduces heparan sulphate biosynthesis | [57,58] |
| *SLC35B2* | Proviral | - Likely functions as a 3'-phosphoadenylyl sulfate: adenosine 3',5'-bisphosphate antiporter at the Golgi membranes. - Mediates the transport of 3'-phosphoadenylyl sulfate (PAPS) from the cytosol into the Golgi lumen. - Provides PAPS, a universal sulfuryl donor, for sulfation reactions occurring within the Golgi compartment. | [59] |
| *MGAT1* | Proviral | - Initiates the formation of complex N-linked carbohydrates. - Plays a critical role in the conversion of high-mannose N-glycans to hybrid and complex N-glycans. | [30] |
