# Supplementary figures and images for: Identification of β4GALNT2 as an anti-hPIV3 factor through genome-wide CRISPR/Cas9 library screening
Source: Emerg Microbes Infect. 2025 Jul 16;14(1):2529895. doi: 10.1080/22221751.2025.2529895 (PMC12269090; doi:10.1080/22221751.2025.2529895)

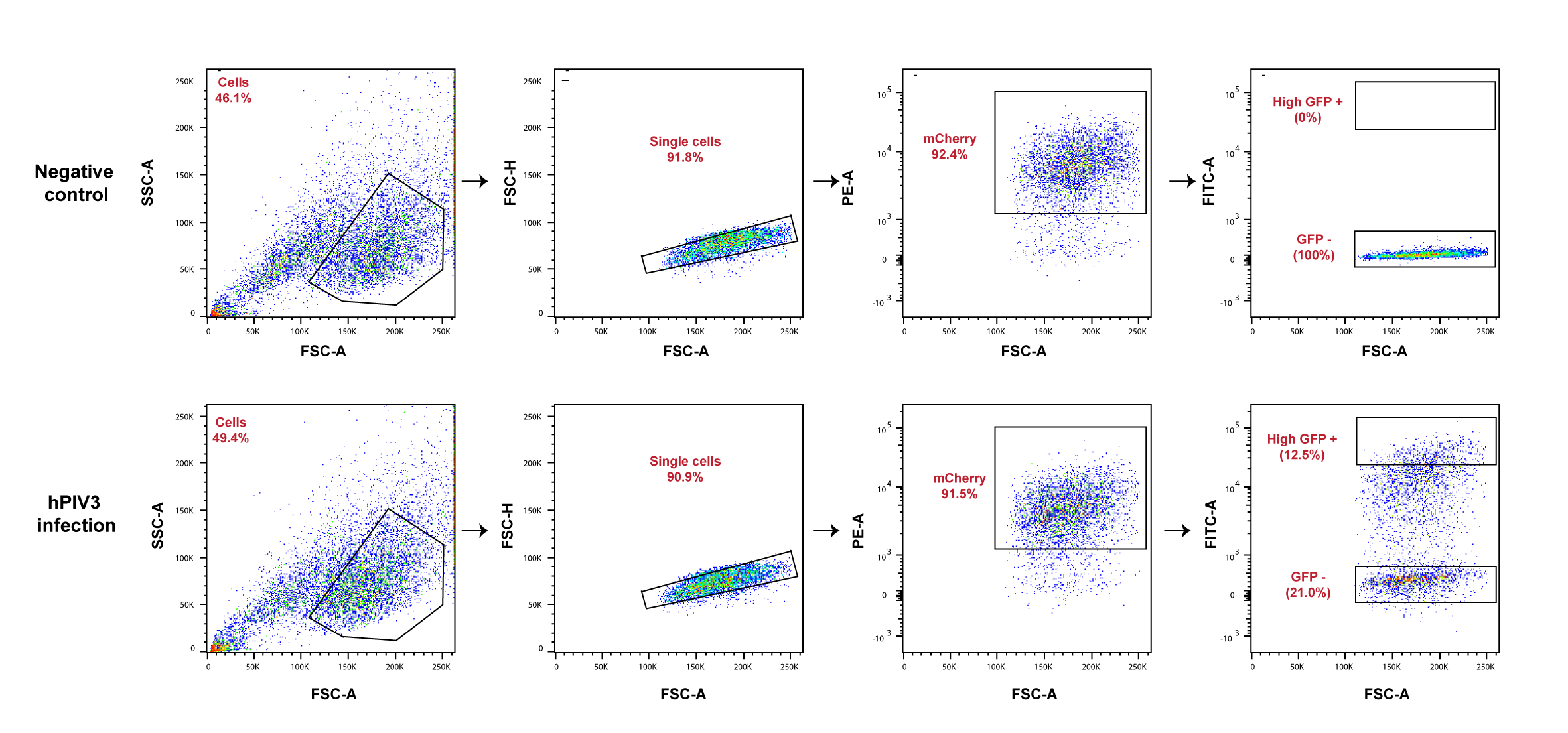

Supplement: Fig S2.tif [file TEMI_A_2529895_SM5466.tif]

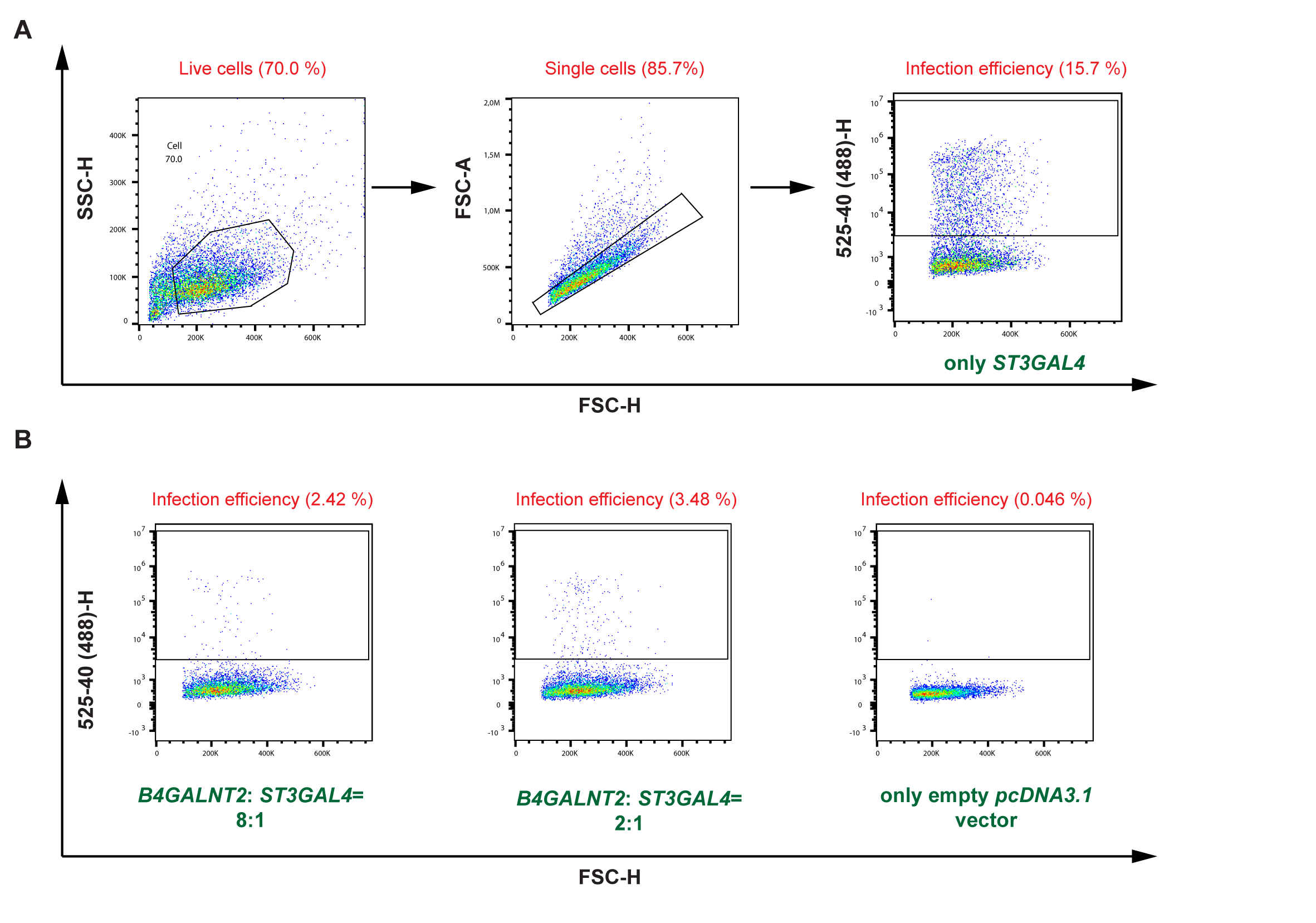

Supplement: Fig S3.tif [file TEMI_A_2529895_SM5465.tif]

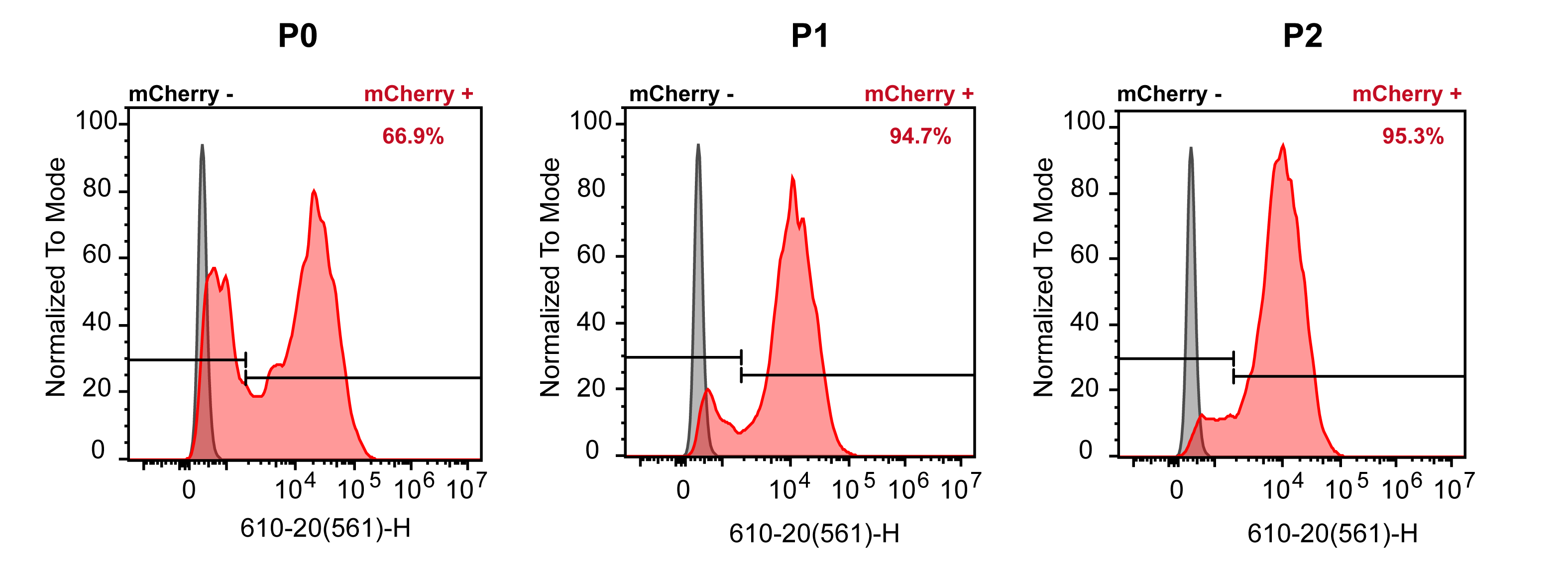

Supplement: Fig S1.tif [file TEMI_A_2529895_SM5463.tif]

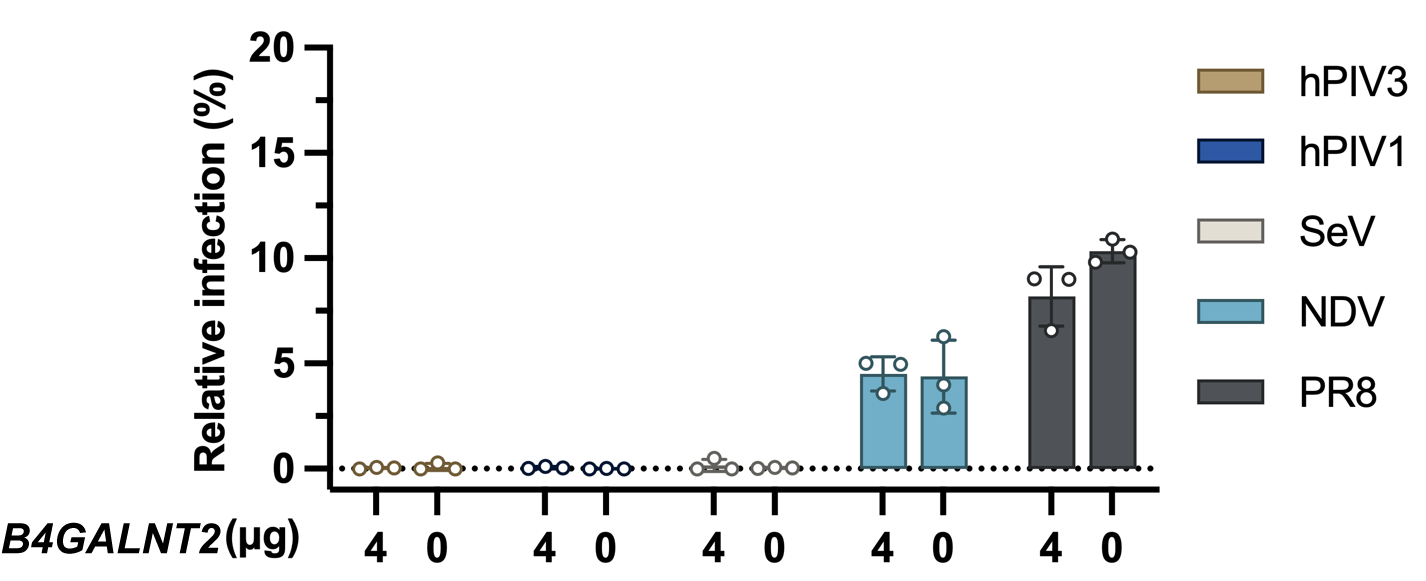

Supplement: Fig S4.tif [file TEMI_A_2529895_SM5462.tif]

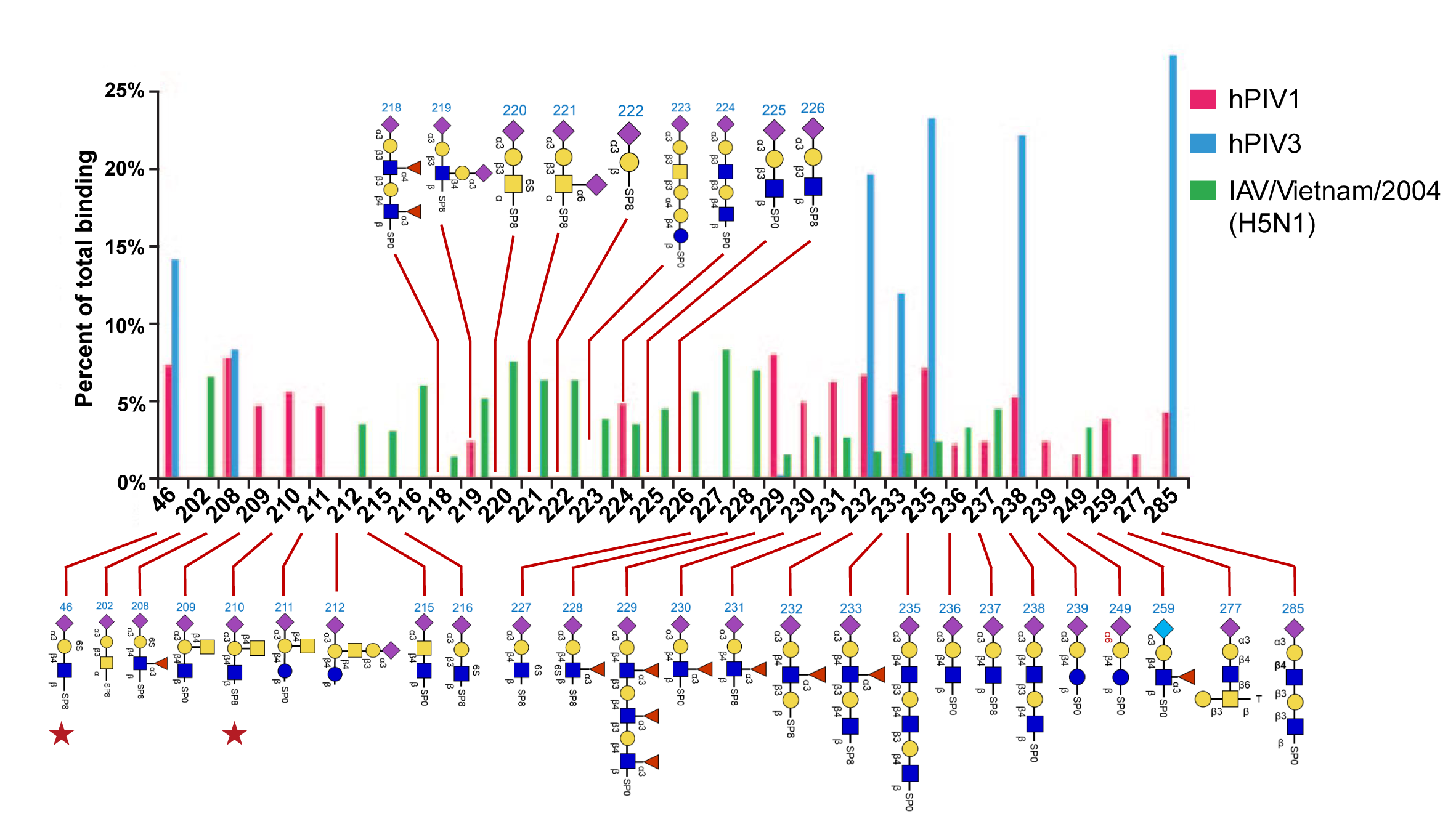

Supplement: Fig S5.tif [file TEMI_A_2529895_SM5459.tif]
